# Comparative transcriptome analysis reveals key genes potentially related to soluble sugar and organic acid accumulation in watermelon

**DOI:** 10.1371/journal.pone.0190096

**Published:** 2018-01-11

**Authors:** Lei Gao, Shengjie Zhao, Xuqiang Lu, Nan He, Hongju Zhu, Junling Dou, Wenge Liu

**Affiliations:** Zhengzhou Fruit Research Institute, Chinese Academy of Agricultural Sciences, Zhengzhou, Henan, China; Huazhong Agriculture University, CHINA

## Abstract

Soluble sugars and organic acids are important components of fruit flavor and have a strong impact on the overall organoleptic quality of watermelon (*Citrullus lanatus*) fruit. Several studies have analyzed the expression levels of the genes related to soluble sugar accumulation and the dynamic changes in their content during watermelon fruit development and ripening. Nevertheless, to date, there have been no reports on the organic acid content in watermelon or the genes regulating their synthesis. In this study, the soluble sugars and organic acids in watermelon were measured and a comparative transcriptome analysis was performed to identify the key genes involved in the accumulation of these substances during fruit development and ripening. The watermelon cultivar ‘203Z’ and its near-isogenic line (NIL) ‘SW’ (in the ‘203Z’ background) were used as experimental materials. The results suggested that soluble sugar consist of fructose, glucose and sucrose while malic-, citric-, and oxalic acids are the primary organic acids in watermelon fruit. Several differentially expressed genes (DEGs) related to soluble sugar- and organic acid accumulation and metabolism were identified. These include the DEGs encoding raffinose synthase, sucrose synthase (SuSy), sucrose-phosphate synthase (SPSs), insoluble acid invertases (IAI), NAD-dependent malate dehydrogenase (NAD-cyt MDH), aluminum-activated malate transporter (ALMT), and citrate synthase (CS). This is the first report addressing comparative transcriptome analysis via NILs materials in watermelon fruit. These findings provide an important basis for understanding the molecular mechanism that leads to soluble sugar and organic acid accumulation and metabolism during watermelon fruit development and ripening.

## 1. Introduction

Watermelon [*Citrullus lanatus* (Thunb.) Matsum and Nakai] belongs to the Cucurbitaceae and is an important and popular staple summertime fresh fruit worldwide. It constitutes 7% of the global area dedicated to fruit and vegetable production [1]. Watermelon fruit provides large amounts of water and nutrients including sugars, carotenoids, lycopene, minerals, and amino acids[2, 3].

Soluble sugars, organic acids, and aroma are important components of fruit flavor and have a strong impact on overall organoleptic fruit quality[4]. Soluble sugars in fruits include fructose, glucose, and sucrose. Malic-, citric-, and oxalic acids are the primary organic acids. The type and content of soluble sugars and organic acids determine the organoleptic properties of fruits. Improvement of fruit quality is an important goal in all watermelon breeding programs. Soluble sugars are important components of watermelon fruit quality and have become the focus of a great deal of research. Previous studies have addressed the dynamic changes in soluble sugar levels and the activities of sugar-metabolizing enzymes that occur during watermelon fruit development and ripening [5–7]. It has been established that the primary soluble sugars in watermelon fruit are fructose, glucose and sucrose[8]. Several quantitative trait loci (QTL) related to sugar content were detected by mapping[9–11], only a Tonoplast Sugar Transporter gene *ClTST2* involved in sucrose accumulation in watermelon fruit was identified by resequencing and biochemical analyses[12], however, sugar accumulation in fruits is a complex quantitative trait, the other key candidate sugar content-regulating genes have not yet been identified. The organic acid composition and content in fruit are also regarded as commercially important traits because they influence organoleptic quality. They also play critical roles in fruit metabolism. Nevertheless, there is relatively little reported research on organic acids in watermelon fruit. It was recognized that malic- and citric acids are the main organic acids in ripe watermelon fruit[13].

Both accumulation and metabolism of soluble sugars and organic acids are developmental stage-dependent. Fruit development and ripening are complex biological processes. They are regulated by several factors including environmental conditions, phytohormones, and gene regulation[14]. Numerous studies have suggested that fruit development and ripening are regulated by the coordinated expression of a set of genes. A total of 832 expressed sequence tags (ESTs) from a subtracted cDNA library of watermelon fruit were utilized to study gene expression as the fruit develops. Of these, 211 were differentially expressed genes with annotation. A significant number were associated with ethylene biosynthesis, transcriptional regulation, pathogen and stress response, carotenoid biosynthesis, and the vascular system[14]. A total of 3,023 differentially expressed genes (DEGs) were identified during watermelon fruit development and ripening, they encoded metabolites related to pigmentation and sweetness[15]. The transcriptome profiles of fruit tissues from cultivated- and wild developing and ripening watermelon were compared. Several DEGs involved in biochemical pathways were identified. These included sugar metabolism and accumulation, flesh carotenoid biosynthesis and metabolism, flesh texture change, ethylene biosynthesis, and signal transduction[16]. Many DEGs related to sugar- and cell wall metabolism, carotenoid biosynthesis, and phytohormone pathways were identified using comparative transcriptome analysis of two different watermelon types during fruit development and ripening[8].

Thus far, there have been no reports on the genes and pathways involved in organic acid metabolism and accumulation during watermelon fruit development and ripening, we have not known that how the soluble sugars and organic acids interconvert each other. In this study, soluble sugars and organic acids content were measured during fruit development and ripening in watermelon, we performed a comparative transcriptome analysis of the watermelon cultivar ‘203Z’ and its near-isogenic line (NIL) ‘SW’. We identified DEGs that may be related to soluble sugar- and organic acid metabolism and accumulation during fruit development and ripening. We confirmed their expression profiles by quantitative real-time polymerase chain reaction (qRT-PCR). These results provide insights into identifying the key candidate genes or pathways involved in soluble sugar- and organic acid metabolism and accumulation during watermelon fruit development and ripening.

## 2. Materials and methods

### 2.1 Plant materials

In this study, the experimental materials included ‘SW’, a near-isogenic line containing foreign introgressed segments from the wild watermelon subspecies ‘PI271769’ of the ‘203Z’ cultivar, and the recurrent parent ‘203Z’. ‘203Z’ and ‘PI271769’ used as germplasm resources were conserved in Zhengzhou Fruit Research Institute. The pure inbred line watermelon cultivar ‘203Z’ has spherical fruits with green rind, dark green stripes, red flesh, a high total soluble sugar content (up to 91.3 mg.g^-1^ FW), and a low total organic acid content (up to 5.90 mg.g^-1^ FW) at maturity. ‘PI271769’ belongs to wild watermelon subspecie, has spherical fruits with white and hard flesh, a low total soluble sugar content (up to 6.83 mg.g^-1^ FW) and a high total organic acin content (up to 17.26 mg.g^-1^ FW). ‘SW’ was derived from a cross between the inbred ‘203Z’ and the wild subspecies ‘PI271769’. The latter has a low total soluble sugar content and a high total organic acid content. The F_1_ plants were backcrossed seven times with ‘203Z’ as recurrent parents to generate BC_7_F_1_ then self-pollinated four times to yield BC_7_F_5_. Progeny with stable high total organic acid content were selected and named ‘SW’. There are no significant phenotypic differences between ‘203Z’ and ‘SW’ except that at maturity, the latter has a higher total organic acid content than the former. In this report, ‘C’ means ‘203Z’ and ‘D’ refers to ‘SW’.

All of the aforementioned experimental materials were grown in a greenhouse in Xinxiang city, China. The female flowers were manually self-pollinated then tagged to record the number of days after pollination (DAP). According to previous studies, red-flesh cultivated watermelon ripens in four critical stages: (1) immature white flesh (C1 and D1); (2) white-pink flesh (C2 and D2); (3) red flesh (C3 and D3); and (4) full-ripe (C4 and D4) (10, 18, 26, and 34 DAP, respectively)[15–17]. Standard conventional field practices (including fertilization, irrigation, and pest control) were followed during the growing season. Twenty four flesh samples were collected from the center of three uniform watermelon fruits at the four development stages then immediately flash-frozen in liquid nitrogen and stored at -80°C until use.

### 2.2 Measurement of the soluble sugar and organic acid content in the fruit pulp

After the watermelon fruit flesh samples were homogenized, their soluble solid content (%) and pH were determined with a laboratory refractometer (HC-112ATC, Shanghai LICHENKEYI, China) and a pH meter (PHB-4, Shanghai LICHENKEYI, China), respectively. The pooled fruit pulp samples were then flash-frozen in liquid nitrogen and stored at -80 °C until they were used to determine the soluble sugar (glucose, fructose, and sucrose) and organic acid (malic, citric, and oxalic) content according to previously reported methods[18].

### 2.3 RNA extraction and quality assessment

Total RNA was isolated from the frozen watermelon flesh using a Plant Total RNA Purification Kit (GeneMark, Beijing, China) according to the manufacturer’s instructions. The quantity, quality, and integrity of the RNA samples were determined with an Agilent 2100 Bioanalyzer (Agilent Technologies, Santa Clara, CA, USA) and a Nanodrop NanoPhotometer (Implen GmbH, Munich, Germany).

### 2.4 cDNA library preparation and sequencing

The cDNA library construction and sequencing were performed at BGITech (Shenzhen, China). The mRNA with polyA tail was enriched with oligo magnetic beads[19], then purified. The cleaved RNA fragments were reverse transcribed to double-strand cDNA using N6 random primer. The cDNA fragments were purified, blunted with phosphate at the 5' end and stickiness 'A' at 3' end, and adaptor-ligated. The ligation product was amplified by two specific primers then denatured by heat. The single-strand DNA was cyclized by splint oligo and DNA ligase. Finally, the cDNA libraries were sequenced on the BGISEQ-500 sequencing platform.

### 2.5 Quality control for raw sequencing and mapping of the reads to the reference genome

The sequences of the adaptor, the unknown bases, and the low-quality reads were removed from the raw reads. The Q20-scores for the clean reads were also calculated. After filtering, the clean reads were stored in FASTQ format[20]. High-quality clean reads were used for downstream analyses. The clean reads were mapped to the reference gene and the genome by Bowtie2[21], and HISAT [22], respectively.

### 2.6 Quantification of gene expression levels and screening differentially expressed genes (DEGs)

Gene expression levels were measured by a software package named RSEM [23]. The FPKM method was used to calculate the expression level using the formula FPKM = [10^9^/NL] C, where C is the number of fragments aligned to the target unigene, N represents the total number of fragments aligned to all genes, and L refers to the length of the target unigene. Differentially expressed genes (DEGs) between two samples were identified using the NOISeq method [24] based on the following default criteria: log2 (fold change) ≥1, and probability of divergence ≥0.8.

### 2.7 GO term and KEGG pathway enrichment

Gene ontology (GO) is a standard international gene function classification system based on molecular functions, cellular components, and biological processes. In this study, the DEGs were annotated by GO using the (http://www.geneontology.org/) database and the 'GO Term Finder' (http://www.yeastgenome.org/help/analyze/go-term-finder). After obtaining the GO annotations for the DEGs, the GO functional classification was executed with WEGO[25]. Genes usually participate in certain biological functions by interacting with each other. To assign the DEGs to specific biological pathways, Kyoto Encyclopedia of Genes and Genomes (KEGG) pathway annotation was used based on the KEGG database[26]. False discovery rates were controlled using methods published earlier[27], setting *P* ≤ 0.05 as a threshold for significantly enriched categories.

### 2.8 Validation of DEG expression by quantitative real-time polymerase chain reaction (qRT-PCR)

Nine differentially expressed genes (DEGs) were selected to validate RNA-Seq output by qRT-PCR. The first-strand cDNA was synthesized using a PrimeScript^TM^ RT reagent kit with gDNA Eraser (Perfect Real Time) (TaKaRa, Kusatsu, Shiga, Japan) based on the manufacturer^’^s protocol. In the first step, genomic DNA contamination was removed from the cDNA by subjecting it to a 10-μL reaction system consisting of 1 μL gDNA Eraser, 2 μL 5XgDNA Eraser Buffer, 5 μL total RNA (200 ng μl^-1^), and 2 μL RNase-free ddH_2_O for 2 min at 42 °C. Then 1 μL PrimeScript RT Enzyme Mix, 1 μL RT Primer Mix, 4 μL 5X PrimeScript Buffer2, and 4 μL RNase-free ddH_2_O were added to the reaction system in the last step. The mixture (final volume 20 μL) was incubated at 37 °C for 15 min followed by 5 s at 85 °C. The qRT-PCR was performed on the LightCycler480 RT-PCR system (Roche Diagnostics International AG, Rotkreuz, Switzerland) using LightCycler 480 SYBR Green I Master (Roche Diagnostics International AG, Rotkreuz, Switzerland) according to the manufacturer^’^s instructions. Gene-specific primers were designed using the database (https://www.ncbi.nlm.nih.gov/tools/primer-blast/). *Cla016178* [28] was used as the internal control gene (S1 Table). Full-length sequences already determined for watermelon[1], were used to design primers. Each reaction system (total volume: 20 μL) contained 2 μL cDNA, 1 μL of each forward- and reverse primer (10 ng mL^-1^), 10 μL 2X SYBR Green real-time PCR Mix, and 6μL ddH_2_O. The PCR program was carried out under the following conditions: initial preheat at 95 °C for 5 min followed by 40 cycles of 30 s at 95 °C, 65 °C, and 72 °C, respectively. Melting temperature curve analysis was performed at the end of each reaction run to confirm the specificity of the qRT-PCR products. Each experiment was performed in triplicate. The raw qRT-PCR data were analyzed with LCS480 v. 1.5.0.39 (Roche Diagnostics International AG, Rotkreuz, Switzerland) and the relative expression levels of the genes were calculated by the 2^-ΔΔCT^ method [29](Livak and Schmittgen, 2001).

## 3. Results

### 3.1 Variations in the soluble sugar and organic acid content during the ripening of ‘203Z’ and ‘SW’ watermelon fruits

**Fig 1:**
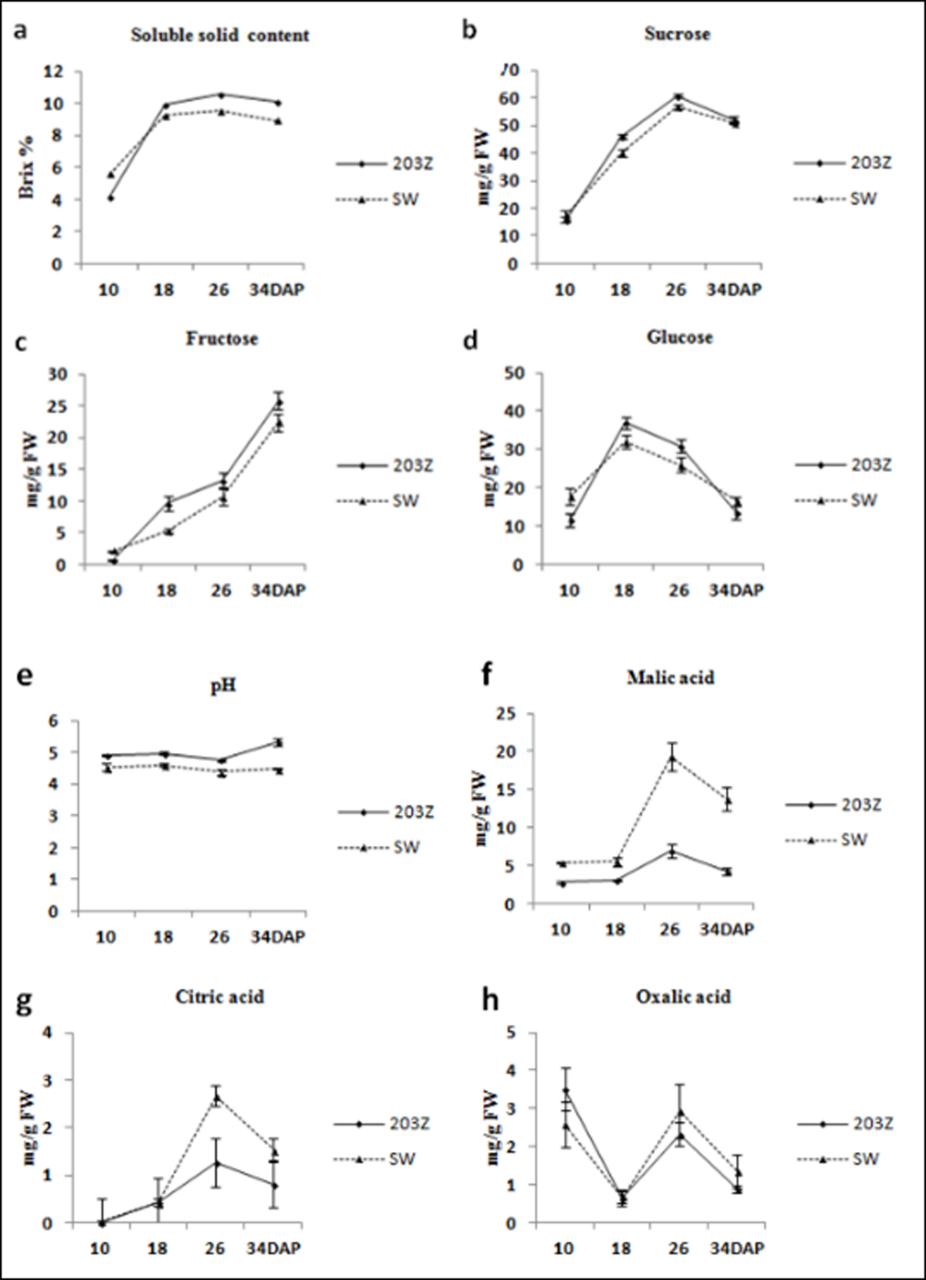
The trends in soluble sugars and organic acids contents in 203Z and SW watermelon fruit during development and ripening. Total soluble sugar (a), fructose (b,) glucose (c), sucrose (d), PH (e), malic acid (f), citric acid (g), and oxalic acid (h) were extracted at 10, 18, 26 and 34 DAP. Three individual replicates were used to reduce the experimental error. The bars represent mean ± SE (n = 3).

The soluble sugar content of watermelon fruit largely determine its quality and the organic acid content influences its flavor. Therefore, the soluble sugar and organic acid content of ‘203Z’ and ‘SW’ fruit were measured during their development and ripening. In watermelon fruit, the dominant soluble sugars are fructose, sucrose, and glucose, and the dominant organic acids are malic acid, citric acid, and oxalic acid. Patterns of change in the soluble sugar and organic acid content during fruit development and ripening in watermelon are shown in Fig 1. In both ‘203Z’ and ‘SW’ fruit, the soluble solid content (SSC) and sucrose content peaked during development at 26 DAP then decreased slightly at 34 DAP (Fig 1A and 1B). Nevertheless, the maximum glucose content was measured at 18 DAP (Fig 1C) while that of fructose was detected at 34 DAP (Fig 1D). Moreover, the SSC, fructose, sucrose, and glucose content in ‘203Z’ were much higher than those in ‘SW’ from 18–34 DAP. The pH of ‘203Z’ and ‘SW’ fruit pulp gradually decreased between 10 DAP and 26 DAP then increased slightly at 34 DAP (Fig 1E). In both ‘203Z’ and ‘SW’, the malic- and citric acid content peaked during fruit ripening at 26 DAP then decreased at 34 DAP (Fig 1F and 1G). The oxalic acid content peaked at the early stage of fruit ripening in both ‘203Z’ and ‘SW’, rapidly decreased to their minimum levels at 18 DAP, increased at 26 DAP, then decreased at the end (Fig 1H). The organic acid contents were significantly lower in ‘203Z’ than in ‘SW’ between 10 DAP and 34 DAP. These results indicate that the soluble sugar and organic acid levels in ‘203Z’ and ‘SW’ fruits significantly differ from each other during development and ripening.

### 3.2 An overview of the RNA-Seq data

To understand the potential molecular synthesis mechanisms involved in the soluble sugar and organic acid of fruit development and ripening, twenty-four cDNA libraries were prepared from fruit flesh samples at the four critical ripening stages. Three biological replicates were used for each stage and watermelon species. After RNA sequencing, the quality of the data was assessed. An overview of the sequencing and assembly is shown in Table 1.

**Table 1:** An overview of the RNA-Seq data.

Sample	Raw reads number	Clean reads number	Total mapped reads(%)	Unique match(%)	Multi-position match(%)^a^	Q20(%)^b^
C1-1	24135583	24119672	97.24	87.67	9.57	97.9
C1-2	24135958	24119571	97.37	87.81	9.56	97.7
C1-3	24136068	24117125	97.88	89.52	8.36	98.0
C2-1	23140509	23122652	98.53	91.27	7.26	98.1
C2-2	23039150	22981291	98.46	91.23	7.23	98.3
C2-3	23235688	23203234	98.52	91.28	7.24	98.2
C3-1	23151242	23116937	98.39	91.06	7.33	98.1
C3-2	23112076	23070091	98.54	91.30	7.24	98.2
C3-3	23223555	23165679	98.51	91.55	6.96	98.4
C4-1	23183013	23145040	98.47	91.06	7.41	98.0
C4-2	23015964	22966485	98.55	91.55	7.00	98.5
C4-3	23716281	23658477	97.49	88.49	9.00	97.5
D1-1	24134726	24113550	97.13	87.12	10.01	97.7
D1-2	24135188	24119312	97.09	87.04	10.05	97.7
D1-3	23386471	23370924	98.48	90.87	7.61	98.1
D2-1	23075487	23032938	98.39	91.04	7.35	98.2
D2-2	22949522	22901119	98.41	91.19	7.22	98.4
D2-3	22792986	22761826	98.41	90.99	7.42	98.3
D3-1	23498245	23484291	98.46	91.29	7.17	98.2
D3-2	23063140	23021956	98.41	91.04	7.37	98.0
D3-3	23135529	23089960	98.39	90.99	7.4	98.1
D4-1	23918095	23865044	97.73	89.09	8.64	97.2
D4-2	23840098	23798619	97.4	88.36	9.04	97.0
D4-3	23814255	23781964	97.7	89.14	8.56	97.6

Notes

^a^ Total Mapped Reads (%) = Unique Match (%) + Multi-position Match (%), are the percentages of clean reads align to reference genome.

^b^ Q20 (%) are the percentages of reads with Phred qualities scores over than 20.

After filtering the low-quality reads, an average of 23,421,989 high-quality clean reads (99.85% of the 23,457,034 raw reads) was obtained. Alignment statistics of reads align to reference genome, in all the samples, >90.1% of the total clean reads from the RNA-Seq data were mapped uniquely to the reference genome whereas only a few (<3%) were not mapped to it. More than 97% of the total clean reads had Phred-like quality scores at the Q20 level. Via comparative transcriptome analysis, this high-quality RNA-Seq data provided a solid foundation for identifying key genes participating in soluble sugar and organic acid syntheses during watermelon development and ripening.

### 3.3 Analyses of differentially expressed genes (DEGs)

**Fig 2:**
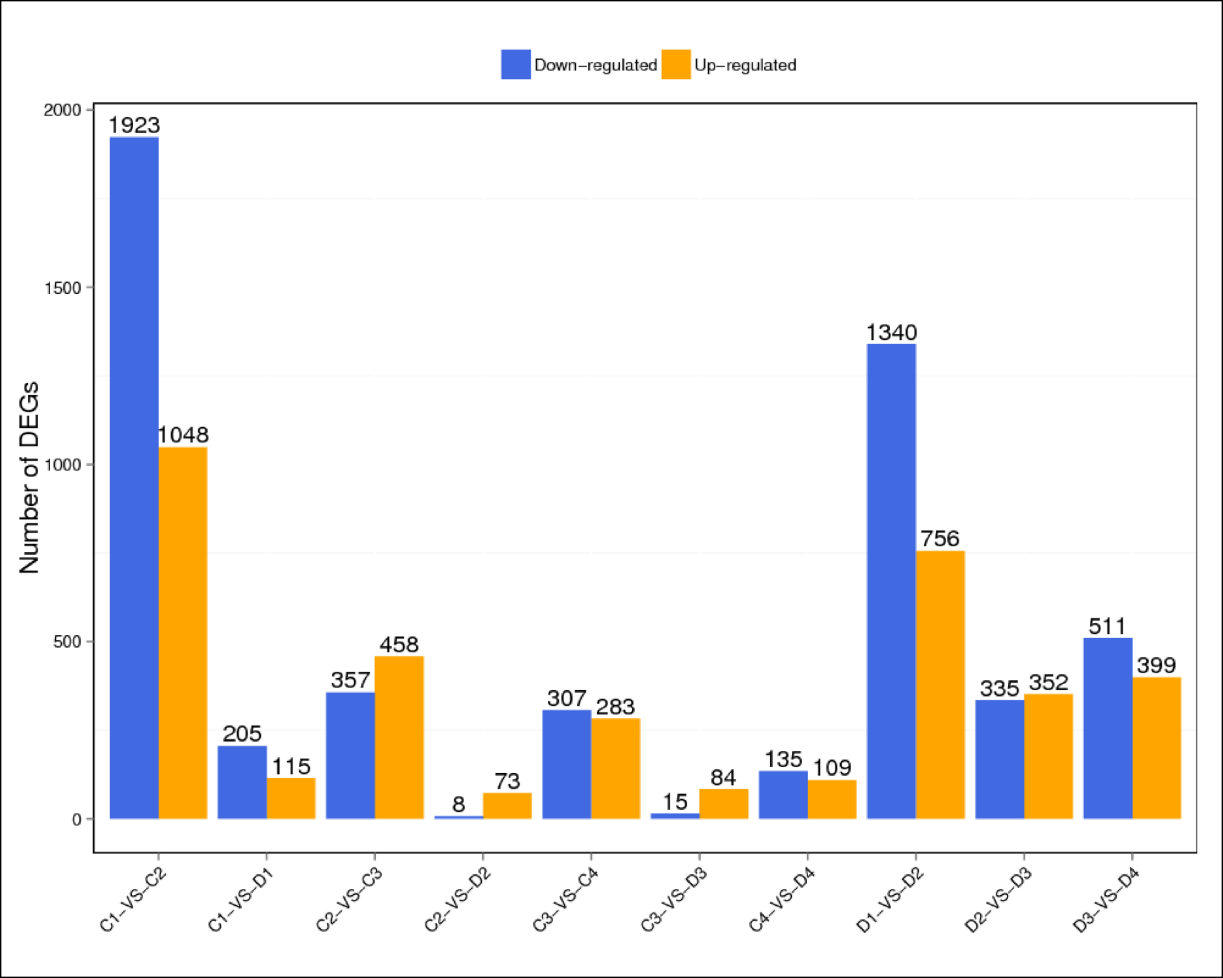
Statistic of differentially expressed genes. X axis represents pairwise and Y axis means number of screened DEGs. Blue bar denotes down-regulated genes and orange bar for the up-regulated genes.

**Fig 3:**
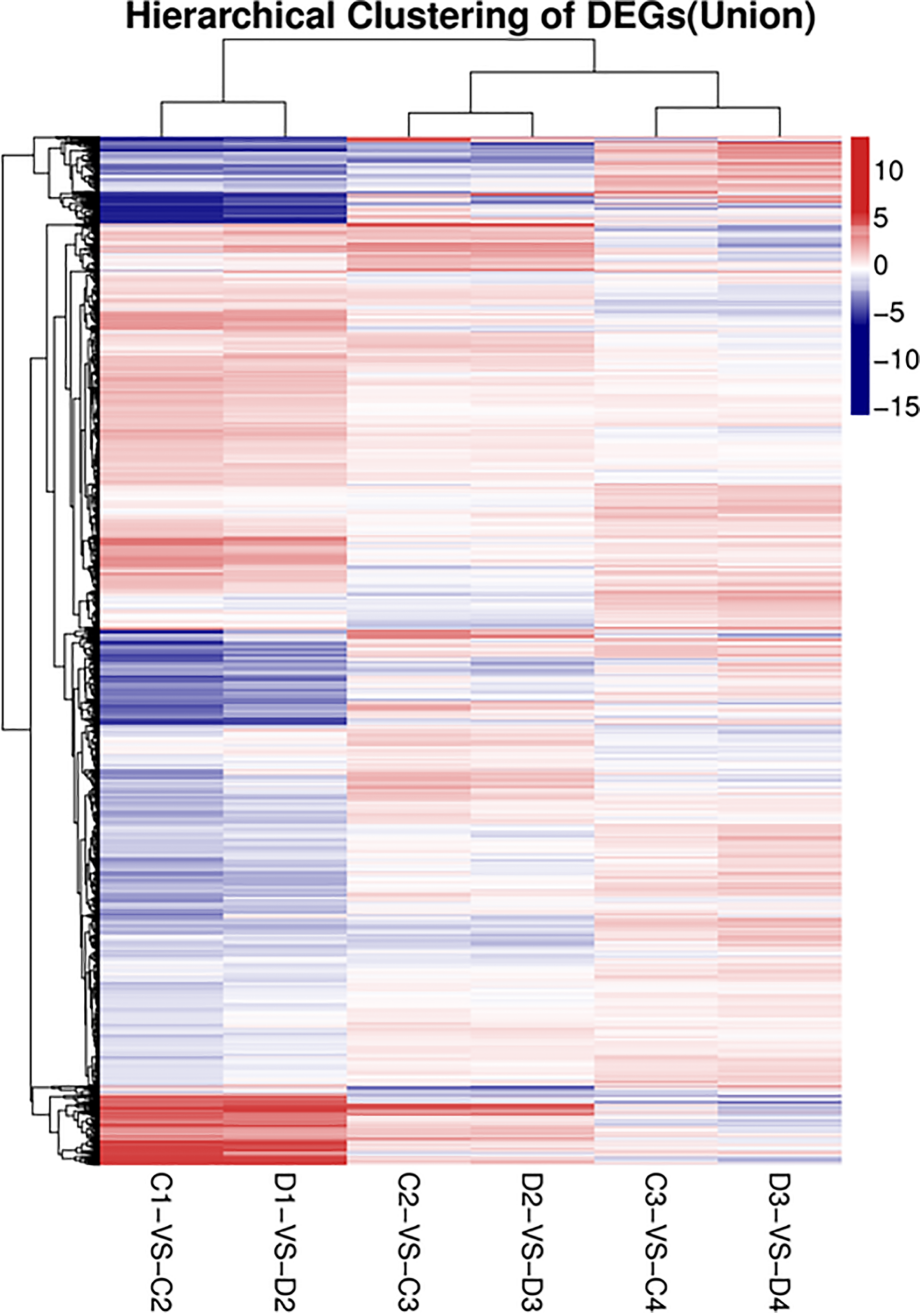
Hierarchical clustering analysis of DEGs between different developmental stages of 203Z and SW. (C1-VS-C2, D1-VS-D2, C2-VS-C3, D2-VS-D3, C3-VS-C4, D3-VS-D4, “a” was the control and “b” was experimental group in “a-VS-b”). Each line refers to data from one gene. The color bar represents the log_2_ (Fold change) and ranges from blue (low expression) to red (high expression).

To find differentially expressed genes between two samples and perform other functional analyses on them, DEG screening was conducted by setting the probability of divergence at ≥0.8 and log2^Ratio^>1 as thresholds. Results are shown in Fig 2. Comparisons of ‘203Z’ and ‘SW’ at the same fruit development time points revealed 320 (C1 vs. D1) and 244 (C4 vs. D4) DEGs. In contrast, C2 vs. D2 disclosed only 81 DEGs and C3 vs. D3 indicated only 99. In the latter case, the numbers of up- and downregulated genes were similar. The analyses of ‘203Z’ and ‘SW’ at different development stages showed that there were far more DEGs in C1 vs. C2 than in C2 vs. C3 or C3 vs. C4. Genes with similar expression patterns often participate in the same biological processes or share functionality. Therefore, hierarchical clustering analysis of the DEG expression patterns was performed (Fig 3). The results demonstrated that there were far more up- and downregulated genes in C1 vs. C2 and D1 vs. D2 than the other groups. The probable reason for this was that the expression level of most genes were low at 10 DAP but, as the fruit developed, most of the genes began to express at high levels in order to drive the increasingly complex biological processes.

After excluding the unigenes (probability of divergence <0.8 or log2^Ratio^<1), a total of 2,260 common DEGs were detected at the various fruit development stages in both ‘203Z’ and ‘SW’. These common DEGs were listed in S2 Table and accounted for 9.6% of the 23,440 predicted genes. There were 636 DEGs identified in ‘203Z’ and ‘SW’ at the same development stages, these DEGs were listed in S3 Table, and they only accounted for 2.7% of the predicted genes. Therefore, transcriptome variation was wider among developmental stages than it was between varieties.

### 3.4 Gene ontology (GO) and pathway functional enrichment analysis of the DEGs

To obtain functional information on the DEGs, a literature search and annotated biological and biochemical functional analyses were run using the WEGO database. Based on the GO classification, the differentially expressed transcripts were classified into three high-level categories: molecular functions, cellular components, and biological processes.

**Fig 4:**
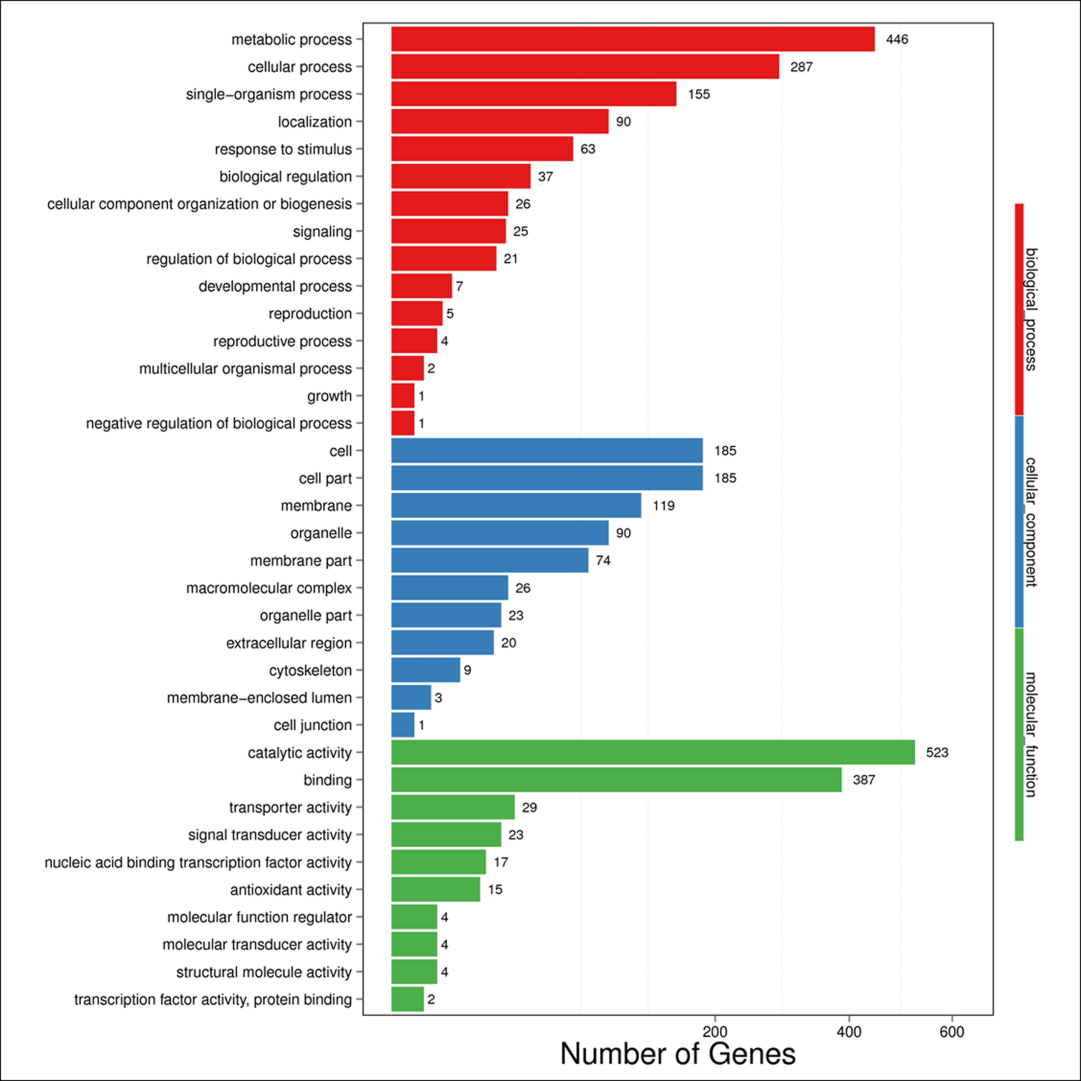
GO functional enrichment analysis for the DEGs in different development stages for the same experimental material. X axis means number of DEGs (the number is presented by its square root value). Y axis represents GO terms. All GO terms are grouped in to three ontologies: blue is for biological process, brown is for cellular component and orange is for molecular function.

**Fig 5:**
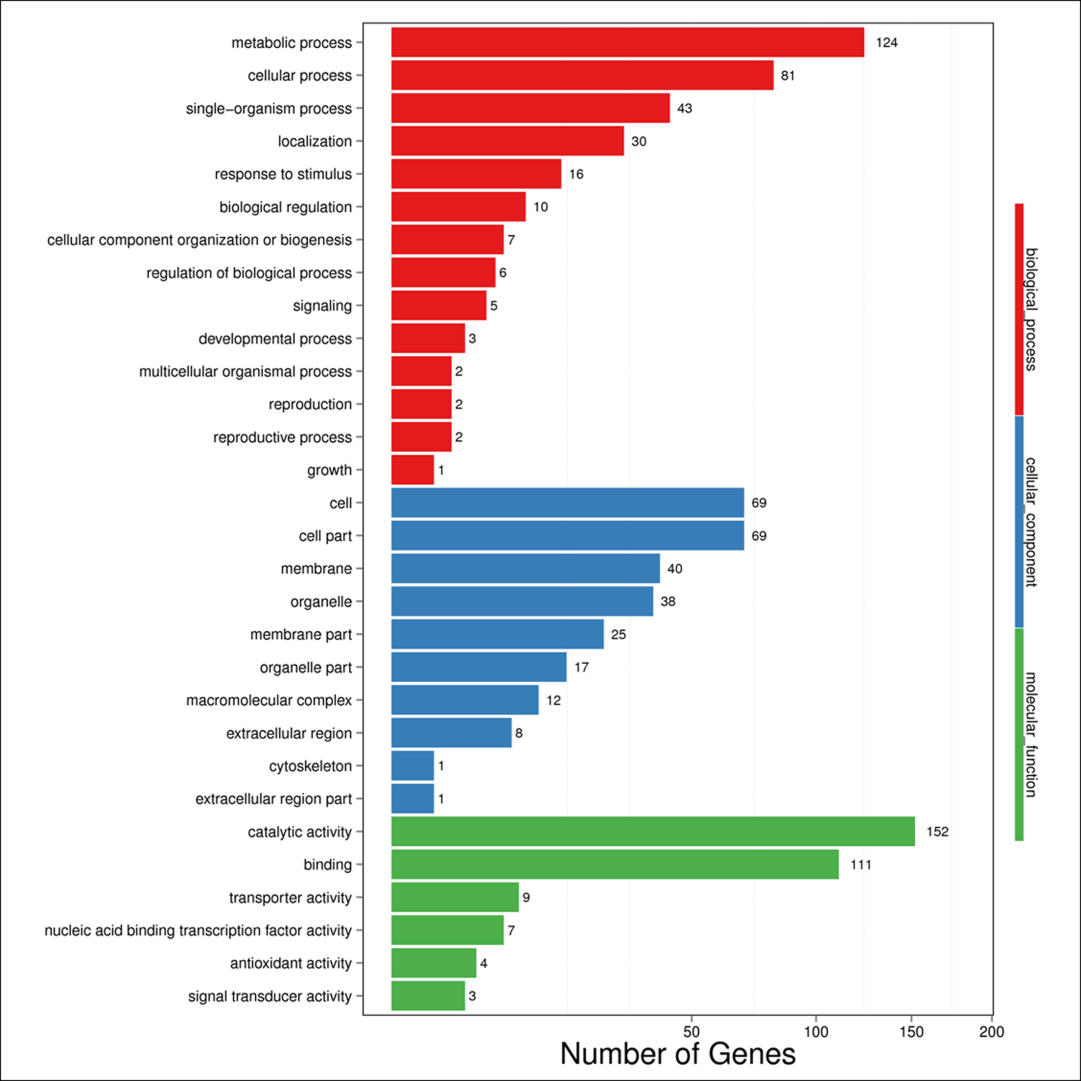
GO functional enrichment analysis for the DEGs in different experimental materials for the same development stage. X axis means number of DEGs (the number is presented by its square root value). Y axis represents GO terms. All GO terms are grouped in to three ontologies: blue is for biological process, brown is for cellular component and orange is for molecular function.

To characterize gene function distribution at the macro level, we performed a GO enrichment analysis to determine the DEG functions at various development stages and in different source materials. The results of the GO functional enrichment are shown in Fig 4 and Fig 5. A total of 848 and 251 unigenes have GO annotations in various development stages (Fig 4 and S4 Table) and different source materials (Fig 5 and S5 Table), respectively. They were grouped into three functional GO categories. For molecular function, “catalytic activity” (523 DEGs for the various developmental stages and 152 DEGs for the different source materials) was the most highly represented GO team, followed by “binding” (387 DEGs for the stages and 111 DEGs for the materials). “Transcription factor activity, protein binding” (only 2 DEGs) was the least represented GO team in the various developmental stages while “signal transducer activity” (3 DEGs) had the least representation for the different source materials. For the cellular component, the categories with considerable enrichment and the highest number of DEGs were “cell” (185 DEGs and 69 DEGs) and “cell part” (185 DEGs and 69 DEGs). Only one team, “cell junction” (1 DEG) was significantly enriched in the various developmental stages. For biological process, the categories with the greatest enrichment and number of DEGs were “metabolic” (446 DEGs and 124 DEGs), followed by “cellular process” (287 DEGs and 81 DEGs) while the “growth” team had only one DEG in this category.

**Fig 6:**
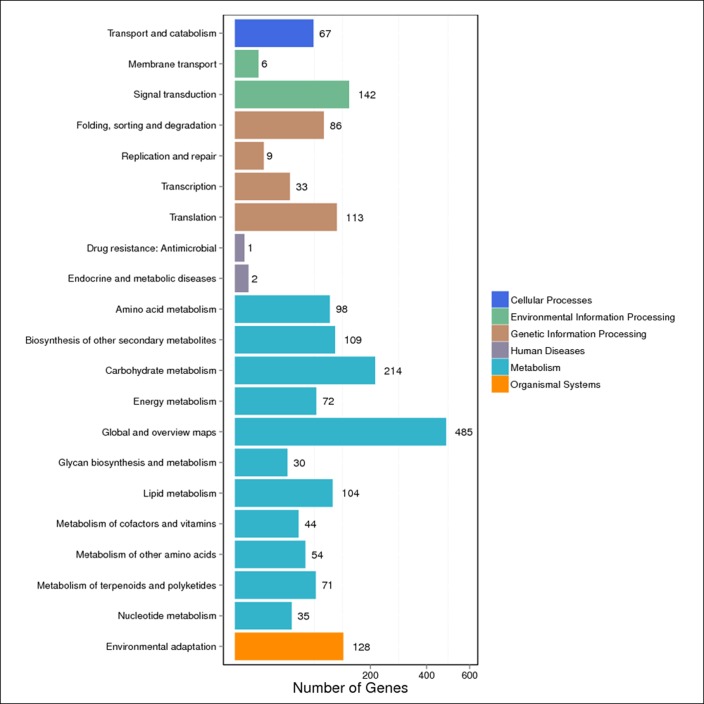
The statistics of KEGG enrichment of the DEGs in different development stages for the same experimental material. X axis means number of DEGs. Y axis represents second KEGG pathway terms. All second pathway terms are grouped in top pathway terms indicated in different color.

**Fig 7:**
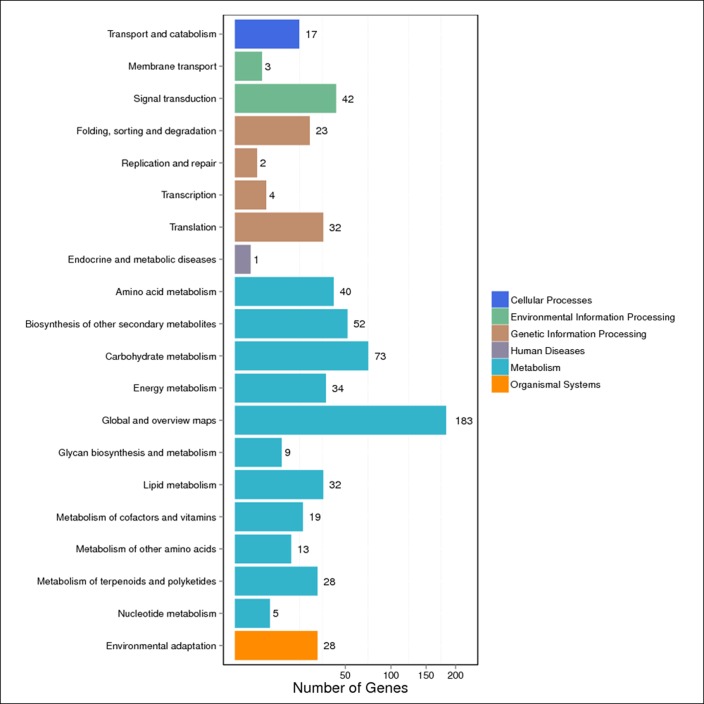
The statistics of KEGG enrichment of the DEGs in different experimental materials for the same development stage. X axis means number of DEGs. Y axis represents second KEGG pathway terms. All second pathway terms are grouped in top pathway terms indicated in different color.

Significantly enriched metabolic pathways and signal transduction pathways in the DEGs were identified by KEGG pathway enrichment analysis. To further identify the biological pathways that are related to the DEGs of fruit development, the detected DEGs were mapped to the reference pathways in the KEGG database and compared to the whole transcriptome background. A KEGG pathway classification and functional enrichment analysis for the DEGs were run in various developmental stages and different source materials. As shown in Fig 6 and Fig 7, 1,620/2,260 DEGs and 458/636 DEGs were mapped to the 20 references pathways in various developmental stages (Fig 6 and S6 Table) and different source materials (Fig 7 and S7 Table), respectively. The pathways with the greatest representation of DEGs were the “global and overview maps” (485 DEGs and 183 DEGs) and “carbohydrate metabolism” (214 DEGs and 73 DEGs). Both of these belong to the “metabolic pathway” whereas only one DEG was mapped to the “antimicrobial resistance” pathway in various developmental stages and one DEG was mapped to the “endocrine and metabolic diseases” pathway in different source materials. In addition, certain DEGs were associated with “transport and catabolism”, “signal transduction”, “biosynthesis of secondary metabolism”, “glycan biosynthesis and metabolism”, “environmental adaptation”, and other pathways. These results indicate that changes in the expression levels of the genes involved in metabolism, signal transduction, and environmental adaptation play critical roles in soluble sugar and organic acid syntheses during fruit development. These annotations provide an important resource for further investigation of the specific pathways involved in watermelon fruit development and ripening.

### 3.5 Expression analysis of the genes related to the soluble sugar and organic acid accumulation and metabolism

Based on our prior knowledge, in watermelon fruit, soluble sugar and organic acid accumulation and metabolism are complex biological processes driven by many different genes. To identify the key genes potentially involved in these metabolic processes, candidates were chosen from the transcriptome data (S8 Table). Several of the DEGs involved in sugar metabolism included genes encoding three sucrose synthases, one encoding sucrose-phosphate synthases, four encoding β-mannosidase, seventeen encoding fructose-bisphosphate aldolase, and four encoding raffinose synthases. Ten genes were involved in organic acid metabolism, including six encoding malate dehydrogenases, one encoding malate synthase, one encoding an aluminum-activated malate transporter, one encoding citrate synthases, and one encoding ATP citrate (pro-S) lyase. In addition, 24.7% of the DEGs were unannotated. These may also participate in soluble sugar and organic acid accumulation and metabolism during watermelon fruit development and ripening.

### 3.6 Validation of differential gene expression data by qRT-PCR

**Fig 8:**
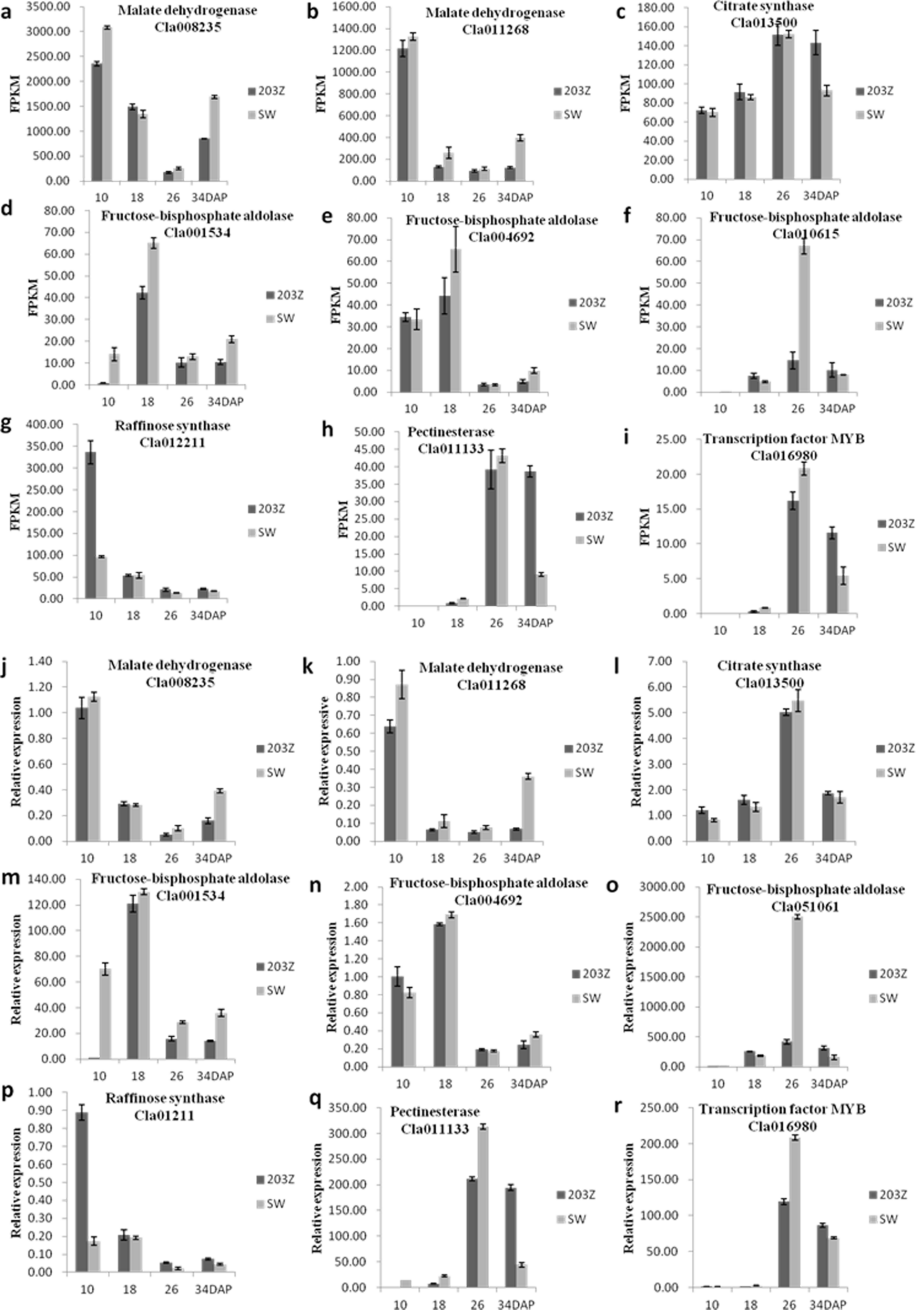
Validation of DEGs by qRT-PCR analysis. The relative expression levels of nine different expressed genes in four development stages of 203Z and SW by RNA-Seq using FPKM method (a-i) and by qRT-PCR using the 2^-△△CT^ method (j-r). Bars represent mean ± SE (n = 3).

To validate the result of the RNA-Seq analysis, nine genes were chosen for qRT-PCR to compare their expression levels among the various samples derived from ‘203Z’ and ‘SW’ fruit during four different development stages. These genes encoded fructose-bisphosphate aldolase (*Cla001534*, *Cla004692*, *Cla010615*), pectinesterases (*Cla011133*), raffinose synthases (*Cla012211*), malate dehydrogenases (*Cla008235* and *Cla011268*), citrate synthases (*Cla013500*), and a regulatory factor (*Cla016980*). The gene expression trends determined by qRT-PCR were consistent with those found in the Seq data, meaning that the transcriptome analysis was reliable (Fig 8).

## 4. Discussion

### 4.1 Innovation of NIL ‘SW’ watermelon with sweet and sour taste

NIL ‘SW’ watermelon with sweet and sour tase was developed in our research, the soluble sugars content was up to 89.71 mg.g^-1^ FW while the organic acids content was up to 19.77 mg.g^-1^ FW at mature stage. Different consumers have different preferences for sweet and sour taste of fruit due to the changes of diet, when the soluble sugars content of fruit is high in different varieties, the variety with high content of the organic acids is more popular [30]. In China, Xinjiang Academy of Agricultural Sciences cantaloupe Research Center has successfully cultivated a series of melon varieties with sweet and sour flavor, which has a broad application prospects. However, the cultivated watermelon varieties are basically sweet varieties all over the world, other varieties with sweet and sour taste are still in the blank period in the market at present. In this study, we first reported ‘SW’ watermelon with sweet and sour taste, the resource can be used as breeding material for research or development of a new breeding or cultivar, which are both attractive to consumers and profitable for growers.

In the present study, we used the watermelon cultivar ‘203Z’ and its NIL ‘SW’ as experimental materials. The latter can accumulate significantly more organic acid than the former during fruit development and ripening. In other respects, however, the two strains do not significantly differences. To the best of our knowledge, this is the first report applying comparative transcriptome analysis to NILs materials in watermelon fruit. The genetic backgrounds of ‘203Z’ and ‘SW’ were highly consistent because of continuous backcrossing and self-pollination. The only genetic difference between them was the presence of foreign introgressed segments from the wild watermelon subspecies. These can significantly reduce background genetic interferences. Therefore, the NILs were ideal experimental materials for comparative transcriptome analysis. At present, many studies using comparative transcriptome analysis for NILs materials have obtained and reported favorable results. Test materials have included cotton[31], wheat[32, 33], rabi sorghum[34], and bell pepper[35].

### 4.2 Dynamic changes of the soluble sugars and organic acids content during fruit development and ripening in watermelon

Soluble sugars and organic acids are important watermelon fruit components influencing organoleptic quality. Sugars are very important determinants of mature fruit quality [36] and are imperative regulatory signals of fruit ripening[37]. In mature ‘203Z’ and ‘SW’ watermelon fruits, fructose, sucrose, and glucose were the major soluble sugars. Sucrose had the highest concentration at ≤52.18 mg/g FW and ≤50.77 mg/g FW in ‘203Z and ‘SW’, respectively. In contrast, the glucose concentrations were the lowest at only ≤13.43 mg/g FW and ≤16.55 mg/g FW in ‘203Z and ‘SW’, respectively. These results were consistent with those obtained for other watermelon varieties in previous studies[7, 8]. Nevertheless, other research has suggested that glucose is the major soluble sugar in mature watermelon fruit[5]. A possible explanation for this discrepancy is the fact that a different watermelon variety was used in that study. The main organic acids found in the mature ‘203Z’ and ‘SW’ watermelon fruit included malic-, citric-, and oxalic acids. These findings corroborate those of the previous report[13]. Overall, the concentrations of soluble sugars of ‘203Z’ were higher and those of organic acids were lower than those of ‘SW’. We predicted that the soluble sugars and organic acids interconvert during watermelon fruit development and ripening. In apple fruit, both the metabolism and the accumulation of soluble sugars and organic acids vary with developmental stage[38, 39]. In the early stages of its development, the fruit accumulates high levels of organic acids but low levels of sugars. At later fruit development stages, however, fructose levels continue to increase while the organic acid concentrations steadily decline. The mechanism by which soluble sugars and organic acids interconvert via glycolysis and the tricarboxylic acid (TCA) cycle is still not completely understood, which need further research.

### 4.3 Genes related to soluble sugar synthesis and metabolism

The sugar content determines the sweetness level of watermelon fruit and is regulated by phloem unloading and metabolism within the fruit flesh[16]. Previous studies have shown that stachyose, raffinose, and sucrose are the main sugars transported in the phloem of cucurbit plants[40, 41]. Raffinose and stachyose are transported to the fruit skin where they are rapidly metabolized[42]. In the present study, differentially expressed genes involved in sugar accumulation and metabolism were identified. The raffinose synthase gene plays an important role in the synthesis of raffinose, *Cla012211*, associated with the raffinose synthase gene, was found to be differentially expressed during fruit development and ripening. It was downregulated and showed similar expression patterns in both ‘203Z’ and ‘SW’, but its expression level was consistently higher in ‘203Z’ than in ‘SW’. Previous studies suggested that the sugars in watermelon fruit were determined primarily by three enzyme families: sucrose synthases (SuSy), sucrose-phosphate synthases (SPSs), and insoluble acid invertases (IAI)[5, 41]. SuSy catalyzes both the synthesis and the hydrolysis of sucrose in plants and is thought to be important in sugar accumulation and metabolism. The gene *Cla009124*, annotated to the SuSy expression level, was downregulated during fruit development in ‘203Z’ and ‘SW’ until 26 DAP at which time its expression level was negatively correlated with sucrose content. This result was not in agreement with the other SuSy genes in watermelon[8, 41]. A possible reason for this discrepancy is that different SuSy genes play distinct roles in sucrose accumulation and metabolism and the processes are reversible. SPSs catalyze sucrose synthesis, and their activity is positively correlated with sucrose accumulation in melon[42], tomato [43] and watermelon[8, 41], which is consistent with our result. *Cla010566* belongs to an ortholog of SPSs and was found to be upregulated at early- then downregulated at mature fruit development stages in ‘203Z’ and ‘SW’. The insoluble acid invertases participate in both phloem unloading and sucrose translocation to fruit skins in sucrose-translocating plants like tomato[19], carrot [44] and watermelon[41]. In our study, *Cla017674* belongs to an ortholog of IAIs and was found to be consistently downregulated during ‘203Z’ and ‘SW’ fruit development and ripening. It was negatively correlated with sucrose content. This result suggests that *Cla017674* may be involved in extracellular sucrose degeneration in watermelon fruit.

### 4.4 Genes involved in organic acid accumulation

Fruit acidity is influenced by organic acid content and is an important component of organoleptic quality. The two main organic acids in most ripe fruits are malic acid and citric acid. Accumulation and metabolism of these acids in the mesocarp cells are closely correlated with glycolysis and the TCA cycle[45]. NAD-dependent malate dehydrogenase (NAD-cyt MDH) is an important enzyme for glycolysis pathway, and can catalyzes the reversible conversion of malate into oxaloacetate (OAA), this step is the most likely route of malate formation[46, 47]. In this study, it was found that *Cla008235* and *Cla011268* belong to an ortholog of NAD-cyt MDH. In ‘SW’, they were downregulated during early fruit development stages, upregulated at maturity, and negatively correlated with malic acid content. The results indicate that *Cla008235* and *Cla011268* participate in malic acid accumulation by watermelon fruit. As is the case with sugars, most of the malate and citrate in fruit is localized in the vacuoles[48]. An aluminum-activated malate transporter (ALMT) may be involved in vacuolar malate transport and accumulation in apple [49, 50] and tomato[51]. *Cla006064* belongs to an ortholog of ALMT and is upregulated early in fruit development then relatively constant at maturity. Its expression level in ‘203Z’ was consistently lower than that in ‘SW’ and highly correlated with malic acid accumulation. This result suggests that *Cla006064* could play a crucial role in increasing fruit malic acid content by facilitating the active transmembrane transport of malate. Mitochondrial citrate synthase (CS) directly controls citric acid synthesis. Previous reports showed that CS activity is positively correlated with citric acid content in citrus fruit [52] and strawberry[53], which corroborates our findings. *Cla013500* belongs to an ortholog of CS. In both ‘203Z’ and ‘SW’, it is upregulated in the early stages of fruit development, downregulated at maturity, and positively correlated with citric acid content. These results indicate that *Cla013500* probably determines citric acid accumulation in watermelon fruit.

## 5. Conclusion

In this study, the soluble sugar and organic acid content were measured and comparative transcriptome analyses were performed on the ‘203Z’ watermelon cultivar and its near-isogenic line (NIL) ‘SW’. To the extent of our knowledge, this is the first report on comparative transcriptome analysis via NILs materials in watermelon fruit. Several DEGs were identified that may be involved in soluble sugar and organic acid accumulation. This discovery improves the understanding of the molecular mechanisms determining soluble sugar and organic acid accumulation and metabolism in watermelon fruit development and ripening.

## Supporting information

S1 Table The primer of genes for qRT- PCR. (DOCX)

S2 Table List of informations of common DEGs among various fruit development stages in both ‘203Z’ and ‘SW'. (XLSX)

S3 Table List of informations of DEGs between ‘203Z’ and ‘SW’ at the same development stages. (XLSX)

S4 Table The GO terms of common DEGs of each main GO category among various fruit development stages in both ‘203Z’ and ‘SW'. (XLSX)

S5 Table The GO terms of DEGs of each main GO category between ‘203Z’ and ‘SW’ at the same development stages. (XLSX)

S6 Table The KEGG pathways of common DEGs among various fruit development stages in both ‘203Z’ and ‘SW'. (XLSX)

S7 Table The KEGG pathways of DEGs between ‘203Z’ and ‘SW’ at the same development stages. (XLSX)

S8 Table The potential key unigenes and pathways involved in soluble sugars and organic acid accumulation and metabolism in watermelon. (DOCX)
