# Supplementary material for: Comparative transcriptome analysis reveals key genes potentially related to soluble sugar and organic acid accumulation in watermelon
Source: PLoS One. 2018 Jan 11;13(1):e0190096. doi: 10.1371/journal.pone.0190096 (PMC5764247; doi:10.1371/journal.pone.0190096)
Supplement: S1 Table — (DOCX) [file pone.0190096.s001.docx]

**Table S1. The primer of genes for qRT- PCR.**

| NO. | Gene ID | Forward primer | Reverse primer |
| --- | --- | --- | --- |
| 1 | Cla008235 | GTTAATCACCAACGTCGCGG | TGCCAGTTTTGACCCCAGTT |
| 2 | Cla011268 | AAGCATCTTCAGGCGTCCTC | GCTCACCATCAGTCACCACA |
| 3 | Cla013500 | CGTTGCTCGTAATCGCTTGG | CAATATTTCAGGCGGCGCAA |
| 4 | Cla001534 | TGATGGGATCAGTTCAGGCG | GTATATGCGGCGGAGAGGAG |
| 5 | Cla004692 | CCAGCTGAGCTCACACTAACA | ACCATAGAGGGCAGCCAAGA |
| 6 | Cla010615 | CGGTGACGTGCTTAATGGGA | GCAAATGCGACGGTGAGAAG |
| 7 | Cla012211 | ATGGCTCAAAAGATGGGCGA | AGGACGACGCTTTTGTGTCT |
| 8 | Cla011133 | TACGAAGACGATTCCGGCAG | TTAATCGAACGCCGTCCTCC |
| 9 | Cla016980 | TTGGTTCCTCAGGCGATGTG | GTCCGGCCAGCAATAACTCT |
| Internal control | Cla016178 | GAACTTGGCACCTGTCCTGT | GAACAGTGCAACAGCCTCAA |
