# Supplementary material for: Comparative transcriptome analysis reveals key genes potentially related to soluble sugar and organic acid accumulation in watermelon
Source: PLoS One. 2018 Jan 11;13(1):e0190096. doi: 10.1371/journal.pone.0190096 (PMC5764247; doi:10.1371/journal.pone.0190096)
Supplement: S8 Table — (DOCX) [file pone.0190096.s008.docx]

**Table S8. The potential key unigenes and pathways involved in soluble sugars and organic acid accumulation and metabolism in watermelon.**

| **Annotation** | **Unigene ID** | **KEGG Orthology** | **Level 1** | **Level 2** |
| --- | --- | --- | --- | --- |
| Sucrose synthase | Cla018637 | K00695 | Metabolism | Carbohydrate metabolism |
|  | Cla017392 |  |  |  |
|  | Cla009124 |  |  |  |
| Sucrose-phosphate synthase | Cla010566 | K00696 |  |  |
| β-mannosidase | Cla013771 | K19355 |  |  |
|  | Cla020782 |  |  |  |
|  | Cla012481 |  |  |  |
|  | Cla014227 |  |  |  |
| Fructose-bisphosphate aldolase | Cla020802 | K01623 |  |  |
|  | Cla016609 |  |  |  |
|  | Cla023160 |  |  |  |
|  | Cla022456 |  |  |  |
|  | Cla018234 |  |  |  |
|  | Cla004692 |  |  |  |
|  | Cla004024 |  |  |  |
|  | Cla018775 |  |  |  |
|  | Cla017433 |  |  |  |
|  | Cla004022 |  |  |  |
|  | Cla004695 |  |  |  |
|  | Cla010387 |  |  |  |
|  | Cla010388 | K02940 |  |  |
|  | Cla022912 |  |  |  |
|  | Cla001534 |  |  |  |
|  | Cla008457 |  |  |  |
|  | Cla004979 |  |  |  |
| Raffinose synthase | Cla012211 | K06617 |  |  |
|  | Cla003446 |  |  |  |
|  | Cla006123 |  |  |  |
|  | Cla019238 |  |  |  |
| Malate dehydrogenase | Cla011268 | K00029 |  |  |
|  | Cla021097 | K00025 |  |  |
|  | Cla020526 | K00051 |  |  |
|  | Cla013314 |  |  |  |
|  | Cla008235 |  |  |  |
|  | Cla004005 |  |  |  |
| Malate synthase | Cla002601 | K01638 |  |  |
| Citrate synthase | Cla013500 | K01647 |  |  |
| ATP citrate-lyase | Cla009244 | K01648 |  |  |
